# Direct Measurement of the Affinity between tBid and Bax in a Mitochondria-Like Membrane

**DOI:** 10.3390/ijms22158240

**Published:** 2021-07-31

**Authors:** Markus Rose, Martin Kurylowicz, Mohammad Mahmood, Sheldon Winkel, Jose M. Moran-Mirabal, Cécile Fradin

**Affiliations:** 1 Department of Physics and Astronomy, McMaster University, Hamilton, ON L8S 4M1, Canada; rosemm2@mcmaster.ca (M.R.); marty@scopesys.ca (M.K.); mahmooma@mcmaster.ca (M.M.); sheldon.winkel@gmail.com (S.W.); 2 Department of Chemistry and Chemical Biology, McMaster University, Hamilton, ON L8S 4M1, Canada; mirabj@mcmaster.ca; 3 Department of Biochemistry and Biomedical Sciences, McMaster University, Hamilton, ON L8S 4K1, Canada

**Keywords:** apoptosis, mitochondria, Bcl-2 family, Bax, Bid, membrane protein, protein–protein interaction, protein oligomerization, fluorescence, single particle detection

## Abstract

The execution step in apoptosis is the permeabilization of the outer mitochondrial membrane, controlled by Bcl-2 family proteins. The physical interactions between the different proteins in this family and their relative abundance literally determine the fate of the cells. These interactions, however, are difficult to quantify, as they occur in a lipid membrane and involve proteins with multiple conformations and stoichiometries which can exist both in soluble and membrane. Here we focus on the interaction between two core Bcl-2 family members, the executor pore-forming protein Bax and the truncated form of the activator protein Bid (tBid), which we imaged at the single particle level in a mitochondria-like planar supported lipid bilayer. We inferred the conformation of the proteins from their mobility, and detected their transient interactions using a novel single particle cross-correlation analysis. We show that both tBid and Bax have at least two different conformations at the membrane, and that their affinity for one another increases by one order of magnitude (with a 2D-$K_{D}$ decreasing from ≃$1.6\;\mu m^{-2}$ to ≃$0.1\;\mu m^{-2}$) when they pass from their loosely membrane-associated to their transmembrane form. We conclude by proposing an updated molecular model for the activation of Bax by tBid.

## 1. Introduction

The regulation step in mitochondrial apoptosis is the permeabilization of the Mitochondrial Outer Membrane (MOM) [1]. It is under the control of the Bcl-2 family of proteins, whose members carry between 1 and 4 Bcl-2 protein homology (BH) regions [2]. The BH3 region, present in all Bcl-2 family proteins, is thought to act as a “death ligand” mediating heterodimerization between family members [3]. Members of the Bcl-2 family have been classified into four different subgroups [2,4,5]. Pro-apoptotic multidomain family members, Bax and Bak, are the executors of the permeabilization process. They oligomerize and form pores in the MOM in response to apoptotic signals. They can be activated by pro-apoptotic BH3-only family members (such as truncated Bid—known as tBid, Bim and Puma [6,7,8]), through a process thought to involve a direct physical interaction [9,10,11]. The Bcl-2 family also includes anti-apoptotic multidomain members (e.g., Bcl-2, Bcl-XL, Mcl-1), which work by either directly inhibiting Bax and Bak, or sequestering BH3-only proteins. The last subgroup in the family is made of the so-called sensitizer proteins (e.g., Bad), which regulates MOM permeabilization by inhibiting anti-apoptotic Bcl-2 family members.

The fate of cells is dictated by the intricate balance of interactions between the different groups of Bcl-2 family proteins, a balance often found to be dysregulated in drug-resistant cancer cells and therefore a sought-after target for pharmacological intervention against cancer [12,13,14,15]. At the centre of the Bcl-2 family interaction network are several competing interactions: the activator-effector interaction (e.g., between tBid and Bax), the repressor-effector interaction (e.g., between Bcl-XL and Bax) and the activator-repressor interaction (e.g., between between tBid and Bcl-XL), each of them able to tip the balance either towards death or survival. Several models have been put forward that try and capture the main features of the activation of Bax, each giving more or less importance to these different interactions, and with the most recent models incorporating the influence of the lipid membrane, increasingly recognized as a main player in the process [16,17,18,19]. One way to discriminate between these different models would be to measure the affinity between different pairs of Bcl-2 family proteins in conditions as close as possible as those encountered in the MOM.

Quantification of the affinity between different pairs of Bcl-2 family proteins has been achieved using ensemble fluorescence resonance energy transfer [10,20,21]. However, it has since become clear that a number of Bcl-2 family proteins (e.g., Bax, tBid, Bcl-XL) exist in a dynamic equilibrium between solution and membrane [22,23,24]. Ensemble measurements, which do not distinguish between the soluble and membrane forms of the proteins, can thus only return an apparent affinity, which depends on the amount and type of lipids present in the system. Interactions between different pairs of Bcl-2 family proteins embedded in the membrane of giant unilamellar vesicles have also been detected using linear scan cross-correlation spectroscopy [25,26,27]. Yet these experiments cannot distinguish between the different conformations or stoichiometries these proteins are known to adopt in lipid membranes [28,29]. In contrast, single particle experiments allow measuring affinities between pairs of proteins with specific conformations and stoichiometries, and have been successfully used by us and others to characterize the oligomerization of tBid [29] and Bax [30] in planar membranes. It is therefore the approach we have chosen here to characterize Bcl-2 family protein interactions. One challenge associated with such measurements is that a low surface density of proteins, typically below the 2D-$K_{D}$, must be used for single particle detection, and therefore interacting protein pairs are rare events—a needle in a haystack. A second issue is that coincidental co-localization events may be observed, leading to an underestimate of the 2D-$K_{D}$ [31]. We solved both issues by combining the ideas of single particle detection and image correlation spectroscopy (building on previous ideas by others [32]), and calculating a single particle cross-correlation coefficient for each detected pair of particles to identify true binding interactions [33].

Here we focus on the activator-effector interaction, using tBid and Bax as a model system. Bid is a BH3-only direct activator of Bax [10,34,35]. It is unique amongst the BH3-only proteins because in solution it adopts a alpha-helical structure packed into a globular fold which is structurally homologous to that of multidomain family members Bcl-2 and Bax [36,37]. Other BH3-only proteins, on the other hand, are known or predicted to be unstructured or to have a structure distinct from that of other Bcl-2 family proteins [38,39,40,41]. Bid gets cleaved by caspase-8 in response to ligand binding to death receptors. The larger of the two resulting Bid fragments, called truncated Bid (tBid), then inserts into the MOM [42]. Upon contact with the membrane, tBid changes conformation and increases its affinity for Bax, which in turn inserts into the MOM [10,43]. Bax then forms oligomers that increase the membrane permeability [6,7]. To fully characterize the interaction between the two proteins at the membrane, we used a reconstituted system capturing all facets of this interaction—full-length fluorescently-labelled purified proteins and a Supported Lipid Bilayer (SLB) with mitochondria-like lipid composition. We used single particle detection on confocal images to sort them into different categories according to their stoichiometry and their mobility, as we have done previously for tBid alone [29], and to detect associations between particles. This allowed us to measure the 2D-$K_{D}$ for each protein subpopulation, which shows the tBid-Bax interaction becomes stronger as the proteins insert deeper into the membrane.

## 2. Results

In order to quantify the affinity between tBid and Bax, we incubated SLB of mitochondria-like composition with purified full-length tBid (labelled with Alexa647) and purified full-length Bax (labelled with HyLite488), at concentrations low enough to achieve single-particle detection. Separate controls were also carried out where the membrane was either incubated with tBid alone or Bax alone. Dual-color confocal image stacks were recorded for each sample, and analyzed in order to extract two-dimensional dissociation constants for different categories of proteins. In the next three sections, we explain how the proteins were detected and classified according to their mobility, how they were further classified according to their stoichiometry, and finally how interactions between tBid and Bax were identified and characterized.

### 2.1. Different Membrane Conformations Are Detected for tBid and Bax Based on Their Mobility

It is well established that tBid and Bax can each adopt different conformations when interacting with a lipid membrane [29]. Proteins with different conformations may have different mobilities, resulting in distinct signatures in both widefield [44] and confocal images [29,45]. We thus looked to classify particles detected in our confocal images according to their mobility.

#### 2.1.1. Signature of Stationary and Mobile Particles in Confocal Images

**Figure 1:**
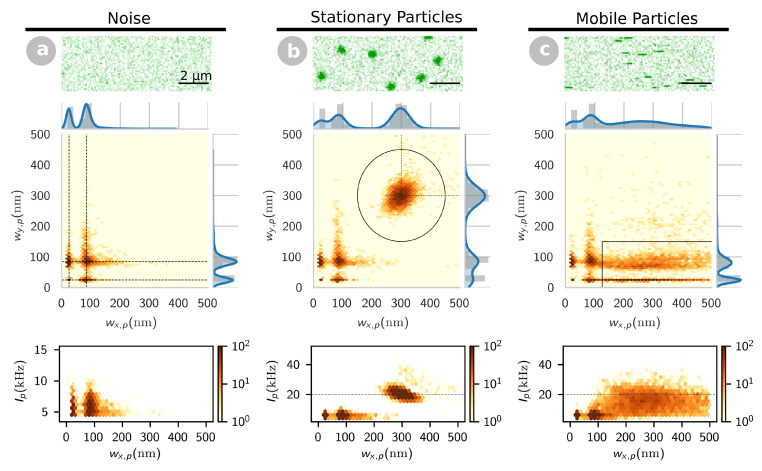
Confocal image simulations for (**a**) background Poisson noise ($i_{B}=1.3$ kHz), (**b**) stationary particles (B=20 kHz) and (**c**) mobile particles ($D=2\;\mu m^{2}\;s^{-1}$, B=20 kHz). For each condition, a single image section is shown as an example (top panel), as well as the particle size distribution (eccentricity map) generated from the analysis of a large number of such images (middle panel), and the relationship between particle width and intensity (bottom panel). In the eccentricity map in (**a**), the dashed lines indicate the position of the two peaks corresponding to spurious photon noise events. In (**b**), the dashed lines show the expected dimensions of an immobile particle ($w_{0}$ both along and perpendicular to the scanning direction), and the circle delimitates the area in which particles are considered “spots”. In (**c**), the rectangle delimitates the area in which the particles are considered “streaks”. In the bottom panels in (**b**,**c**), the dashed line indicates the value of the molecular brightness, *B*.

We performed simulations to establish the link between particles’ apparent shape in confocal images and their mobility. We simulated the two-dimensional diffusion of particles with varying mobilities and molecular brightness, and the acquisition of confocal images using conditions similar to those typically used in our experiments (confocal detection volume radius $w_{0}=300$ nm, pixel size d=100 nm, pixel dwell time δ=1 ms). Examples of simulated images for both completely stationary particles and diffusing particles ($D=2\;\mu m^{2}\;s^{-1}$) can be seen in Figure 1. Events can easily be detected in both cases, but their appearance is strikingly different: diffraction limited spots for stationary particles and single line streaks along the scanning direction for diffusing particles (as previously observed for both proteins and lipids in SLB [29,46]).

The single particle detection procedure described in the Methods section was applied to multiple series of simulated images of either stationary or diffusing particles (noise images were also analyzed for comparison). For each detected event, this procedure returns the radii of the 2D-Gaussian spot that best fit the particle (Equation (4)), $w_{x,p}$ along the scanning direction and $w_{y,p}$ perpendicular to it, as well as the intensity of the particle, $I_{p}$. By inspecting the eccentricity maps (two-dimensional distributions of particle radii, shown in Figure 1), multiple clusters can be identified. In images containing only background photon noise (Figure 1a), only single pixel events concentrated in a small region centred around $w_{x,p}\approx w_{y,p}\approx d=100\;nm$ are detected, as expected since photon noise is not spatially correlated. (There is both a horizontal gap and a vertical gap around ≈50 nm, the result of the instability of the Gaussian fitting of a signal detected in a single pixel.) Stationary particles (Figure 1b) appear as a cluster of diffraction-limited events around $w_{x,p}\approx w_{y,p}\approx w_{0}=300\;nm$ and $I_{p}\approx B=20\mathrm{kHz}$. They are well separated, both by their dimensions and intensity, from events due to noise, also detected in this case. We thus decided to classify events as stationary particles if they were found within a distance $w_{0}/2$ of their expected position $\left(w_{0},w_{0}\right)$ on the eccentricity map. For diffusing particles (Figure 1c), the eccentricity map shows an extended cluster of events with $w_{y,p}<w_{0}/2=150\;nm$ and $I_{p}$ around or below B=20kHz. The distinction between diffusing and stationary particles, based on their shape, is therefore straightforward in this case. However, the distinction between noise and diffusing particles is not, as the distributions of these two types of events overlap, both on the eccentricity map and on the intensity map. As a compromise between false positive and false negative detection, we decided to classify events as mobile particles only if $w_{y,p}<w_{0}/2$ and $w_{x,p}>1.2\;d=120\;nm$. In these conditions, noise events only contribute a maximum of 10% of the mobile particle detection events, which was judged acceptable.

**Figure 2:**
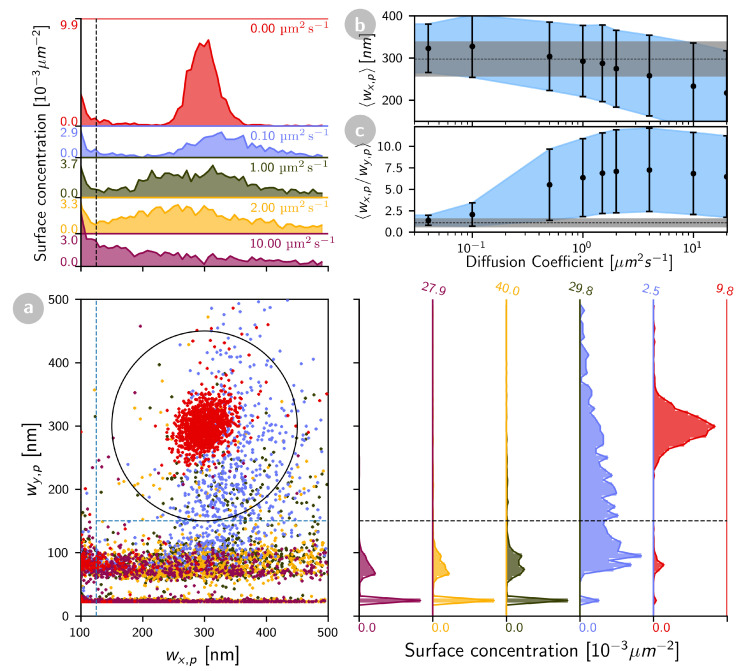
Influence of particle diffusion coefficient on the appearance of events detected in simulated confocal images. Simulation parameters were otherwise the same as in Figure 1. (**a**) Distribution of $w_{x,p}$ and $w_{y,p}$ values for particles with different diffusion coefficients (red: $0\;\mu m^{2}\;s^{-1}$, blue: $0.1\;\mu m^{2}\;s^{-1}$, green: $1\;\mu m^{2}\;s^{-1}$, yellow: $2\;\mu m^{2}\;s^{-1}$, purple: $10\;\mu m^{2}\;s^{-1}$). (**b**) Average streak length and (**c**) average eccentricity of all events with $w_{x,p}>120$ nm (error bars are the standard deviation) as a function of diffusion coefficient. The dashed line and the grey area represents the values obtained for stationary particles.

To further characterize the mobility of the diffusing particles, we considered the length and eccentricity of the streaks, which depend on the particles’ trajectory during the imaging, and therefore on their mobility [47]. Simulations (Figure 2) show that the average streak length, $\langle w_{x,p}\rangle$, decreases as *D* increases, while the average eccentricity, $\langle w_{x,p}/w_{y,p}\rangle$, first increases from 1 to $≃2w_{0}/d=6$, then decreases. For $D>20\;\mu m^{2}\;s^{-1}$, particle events become difficult to separate from noise events, as the lengths of the streaks shorten. The time needed to image a full line in the confocal image is $n_{x}\delta =0.1$ s in the conditions of our experiments, thus the time necessary to capture a stationary particle is $≃n_{x}\delta \times w_{0}/d=0.3$ s. The cut-off between particles appearing as “spots” and “streaks” should occur when they are able to diffuse over a distance $w_{0}$ during that time, in other words when $D>D_{c}=w_{0}^{2}/\left(4n_{x}\delta \times w_{0}/d\right)=0.08\;\mu m^{2}\;s^{-1}$. This is indeed what is observed in our simulations (Figure 2a). Thus, the classification method introduced here allows, in the conditions of our experiments, sorting events into “stationary” proteins ($D≲0.1\;\mu m^{2}\;s^{-1}$) and “mobile” proteins ($D≃0.1-20\;\mu m^{2}\;s^{-1}$).

#### 2.1.2. tBid and Bax Mobility in the Mitochondria-Like SLB

**Figure 3:**
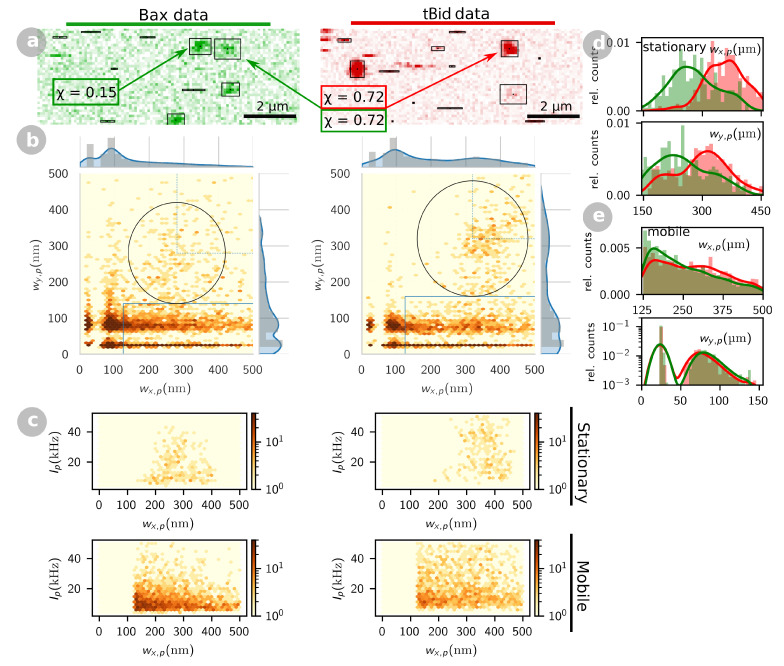
Event detection for confocal images of SLB incubated with tBid-Alexa647 and Bax-HyLite488. (**a**) Representative example of a section of a pair of confocal images (left: green detection channel, right: red detection channel) acquired for a SLB incubated successively with 0.2 nM tBid-Alexa647 and 1.0 nM Bax-HyLite488. Black boxes highlight all the events detected in these images. (**b**) Eccentricity maps and (**c**) intensity maps obtained for the entire set of 258 images acquired (regardless of Bax concentration). Solid lines delimitate the regions used to classify the particles as stationary or mobile, considering $w_{0}=280\mathrm{nm}$ (green channel) or $w_{0}=320\mathrm{nm}$ (red channel). The distributions of observed $w_{x,p}$ and $w_{y,p}$ values are given in (**d**) for stationary particles and in (**e**) for mobile particles (green lines and bars: Bax, red lines and bars: tBid).

We applied the single particle detection and classification method outlined in the previous section to a set of 258 dual-color confocal images of SLB incubated successively with 0.2 nM tBid-Alexa647 and Bax-HyLite488 at concentrations ranging from 0.1 to 2 nM (see Methods for experimental details). An example of a pair of such images (green and red detection channels) can be seen in Figure 3a, where detected events are marked with a box (a number of detection events are rejected, either because they are too close to the border or to another already detected event, because they cannot be correctly fitted by a Gaussian, or because they have an intensity below the set threshold intensity). In these images, both spots and streaks are observed, indicative of the presence of both stationary and mobile proteins. Accordingly, the corresponding eccentricity maps show particles both in the mobile and stationary particle regions (Figure 3b). The number of particles detected per frame varied only slightly across conditions, and was roughly the same for both proteins (SI Figure S2). In each channel, we detected on average ≃5 to 10 mobile particles per frame, significantly more than stationary particles, found at a rate of ≃1 to 3 particles per frame. Even including poorly defined particles rejected from the final analysis, the total number of detected particles remained on average below 30 per frame and per channel, i.e., below a concentration of 0.3 particles/$\mu m^{2}$. For both Bax and tBid, our data thus indicates the presence of at least two different types of membrane conformation—an abundant highly mobile form and a rarer stationary form. When the same experiment was repeated with tBid alone, both mobile and stationary particles were also observed in the membrane, as we already reported in Ref. [29]. In contrast, when the experiment was repeated with Bax alone, no particles could be detected at the membrane (even at the highest 2 nM Bax concentration used for incubation, see SI Figure S3), highlighting the important role played by tBid in retaining Bax at the membrane in this reconstituted system.

Stationary Bax-HiLyte488 have an average apparent radius $\langle w_{x,p}\rangle =\left(280\pm 60\right)\;\mathrm{nm}$ (mean ± stdv), 10 to 20% smaller than that of stationary tBid-Alexa647 ($\langle w_{x,p}\rangle =\left(360\pm 50\right)\;\mathrm{nm}$), as expected given the difference in excitation and detection wavelengths between the green and red channels, and in each case close to the radius of the confocal observation volume measured by FCS. Of note, the distributions of values for $\langle w_{x,p}\rangle$ and $\langle w_{y,p}\rangle$ obtained for stationary Bax and tBid (Figure 3d), while clearly belonging to a well-defined population, are more spread out than that obtained in simulations (Figure 1b), and the average eccentricity of these particles is slightly above 1 ($\langle w_{x,p}/w_{y,p}\rangle =1.1$ for Bax and 1.2 for tBid). Thus, “stationary” tBid and Bax particles might include particles with a small mobility, on the order of 0 to $0.1\;\mu m^{2}\;s^{-1}$ (other effects, such as photobleaching, could also explain this slight eccentricity).

For mobile particles, the distribution of detected shapes, as seen in the eccentricity maps (Figure 3b), and the distributions of $w_{x,p}$ and $w_{y,p}$ values (Figure 3e), are also as expected from the simulations. From the average length of these streaks ($\langle w_{x,p}\rangle =250\;\mathrm{nm}$ for Bax and 280 nm for tBid) and their average eccentricity ($\langle w_{x,p}/w_{y,p}\rangle =4.4$ for Bax and 4.9 for tBid), which can be compared to that obtained for simulated particles with different mobilities (Figure 2), we conclude that the mobile membrane fractions of both tBid and Bax have diffusion coefficients on the order of 10 to $20\;\mu m^{2}\;s^{-1}$, corresponding to protein configurations only loosely associated with the membrane (since they are faster than lipids, which in SLB have diffusion coefficients in the 1 to $5\;\mu m^{2}\;s^{-1}$ range [48,49,50]).

### 2.2. tBid and Bax form Oligomers at the Membrane

**Figure 4:**
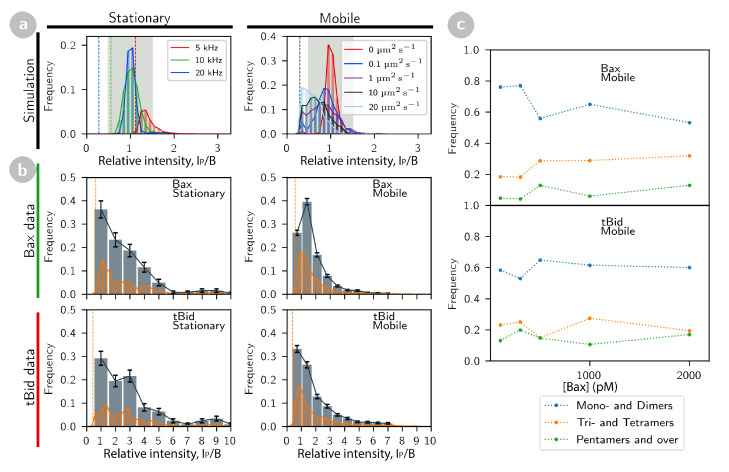
Intensity distribution and stoichiometry of detected events. (**a**) Intensity distributions for events detected in simulated confocal images for either stationary particles with different brightness (left panel) or particles with brightness B=20 kHz and different diffusion coefficients. (**b**) Intensity distributions for events detected in confocal images of SLB incubated with tBid-Alexa647 and Bax-HyLite488, classified for each protein into stationary and mobile particles (regardless of Bax concentration). All intensities have been normalized by the known particle brightness of the monomer. The experimental distributions are binned in two different ways, with a small bin width of B/4 for visualization (orange bars) and with a larger bin width of *B* or 0.7B for stationary and mobile particles, respectively, representing the expected apparent brightness of a monomer for these two types of particles (blue bars). The coarse binning thus provides an estimate of the relative abundance of different protein stoichiometry. In (**a**,**b**), the dashed vertical bars indicate the threshold intensity used for particle detection. (**c**) Oligomer frequency versus Bax-HyLite488 concentration (for a constant 0.2 nM tBid-Alexa647 concentration).

Because of photon noise, and because of eventual motions during imaging, the intensity of an event may not be equal to the particle molecular brightness (see Figure 1b,c, bottom panels). Simulations of stationary particles for different values of the brightness *B* (and at a concentration of 20 particles per image, comparable to those encountered in our experiments), do show that the distribution of intensities for detected particles is usually quite narrowly peaked around *B* (Figure 4a). For low intensity particles, for which the fixed detection threshold becomes on the order of *B*, the intensity distribution becomes broader and is centred slightly above *B* (Figure 4a, left panel). However, as the mobility of the particles increases, the centre of the distribution shifts from *B* towards lower values (Figure 4a, right panel). In stark contrast, the distribution of intensities for recorded images of tBid and Bax is very broad, in some cases with visible peaks at intensities that are much larger than *B* (Figure 3c and Figure 4b), as was previously observed for both proteins separately [29,30]. These extended distributions are consistent with the presence of oligomers, with intensities representative of the number of monomers it contains (barring issues with incomplete labelling or photobleaching). When comparing the size distribution of the tBid oligomers observed in the presence of Bax (Figure 4b, lower panels) with that observed in the absence of Bax (as reported in Ref. [29]), the only visible difference is a slight shift of the distribution towards smaller size oligomers in the presence of Bax.

We first concentrate on the distribution of intensities for stationary tBid and Bax (Figure 4b, left panels, in which the results of all our experiments are congregated regardless of Bax concentration). Detected event intensities have been normalized by the known intensity of the monomer, which was separately measured before each experiment using fluorescence correlation spectroscopy (FCS), and found to be on average 〈B〉=13kHz for cBid-Alexa647 and 〈B〉=7.6kHz for Bax-HyLite488. For both tBid and Bax, we see a peak around a normalized intensity of 1 (corresponding to particles with brightness *B*) followed by a series of harder to distinguish peaks going up to a normalized intensity of about 10. Intensity bins of width 1 and centred on integer values (corresponding to B=1, 2, etc.) were used to sort the data into putative monomers, dimers, etc. Although this classification is not very precise (because of day-to-day variation in the value of *B*, incomplete labelling and photobleaching), it allows us to conclude that, for both proteins, there is a broad distribution of stationary oligomers up to the tetramers, with rarer oligomers as large as decamers.

The distribution of intensities for mobile particles is noticeably different than that of stationary particles, with a shorter tail at higher intensities (Figure 4b, right panels). Since these particles have a diffusion coefficient around 10 to $20\;\mu m^{2}\;s^{-1}$, our simulations show that an oligomer with brightness nB should have an intensity around 0.7 nB (Figure 4a, right panel). The abundance of different mobile oligomers was therefore estimated by binning the distributions of normalized intensities into bins of width 0.7 centred around multiples of 0.7. For both tBid and Bax, the proportion of low stoichiometry detections is higher for mobile particles than for stationary particles. This is true even if, for mobile particles, the intensity threshold (≃B/2) is close to the centre of the monomer bin, meaning that a significant number of mobile monomers must go undetected. This suggests that oligomer immobilization in the SLB, which is probably due to deeper insertion into the lipid bilayer, tends to be associated with larger stoichiometries (in agreement with previous observations for tBid [29]).

Looking at relative oligomer abundance as a function of the concentration of Bax incubated with the SLB (Figure 4c), and focussing on the mobile particles which are more abundant and for which we have better statistics, shows that in all the explored experimental conditions, Bax was active enough and concentrated enough to form oligomers with a size (tetramer) generally considered large enough to constitute a pore [51]. A slight decrease in the concentration of low stoichiometry Bax oligomers (monomers and dimers), at the profit of larger size oligomers, can be seen when Bax concentration is increased (no discernible change in oligomer composition can be detected for tBid).

### 2.3. Quantification of the Interaction between tBid and Bax

Two-channel confocal images hold information about potential molecular interactions via the colocalization of events detected in both channels. A difficulty in positively identifying interactions is that particles might coincidentally be located within the same pixel. To avoid this issue, we have previously proposed the use of a quantity called the particle cross-correlation coefficient, χ, calculated as the spatial cross-correlation between the fluorescence intensity fluctuations recorded in both channels over a small region around the particle (see the Methods section for details) [33]. For perfectly co-localized particles, we expect χ=1, while for distant particles χ=0. Here we explore the sensitivity of χ on the distance between particles through simulations, before using it to detect interactions between tBid and Bax particles.

#### 2.3.1. The Particle Cross-Correlation Coefficient Decreases with the Distance between Particles

**Figure 5:**
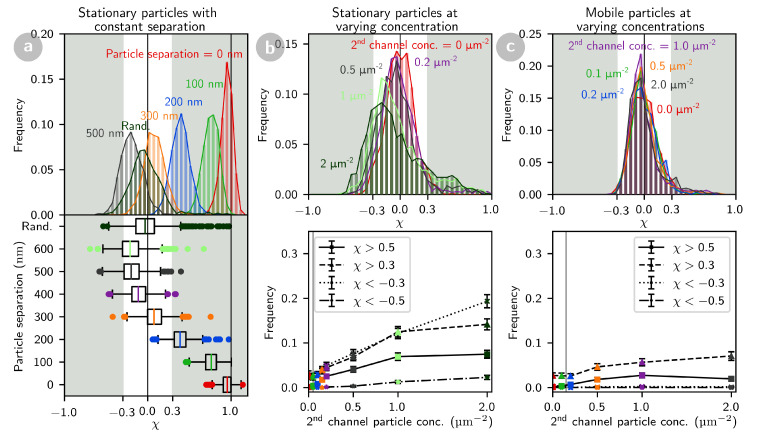
Distribution of particle cross-correlation coefficients obtained for simulated two-channel confocal images. (**a**) Particle cross-correlation distribution for particles separated by a fixed distance Δr. (**b**,**c**) Effect of increasing the particle concentration in the second channel for randomly placed particles, either stationary (**b**) or mobile (**c**). The lower panels in (**b**,**c**) give the frequency of accidentally correlated (χ>0.3 and χ>0.5) and anti-correlated (χ<−0.3 and χ<−0.5) events. All distributions show the cross-correlation of the particles detected in the green channel. Grey vertical lines in (**b**,**c**) lower panels indicate the experimental conditions.

Particle tracks and dual-channel confocal images were simulated for pairs of particles (one in the green detection channel, with $w_{0,g}=320\;\mathrm{nm}$, and one in the red detection channel, with $w_{0,r}=370\;\mathrm{nm}$). The position of the two particles was either completely independent or offset by a fixed value Δr, and the particles were either mobile or stationary. For each condition, 100 pairs of confocal images (each containing 14 to 20 particles) were generated. Images were analyzed as described above, and for each detection event in the first channel, χ was calculated (Equation (5)). For exactly co-localized particles (Δr=0), the obtained values of χ are peaked around 1, but when Δr is increased, the average value of χ decreases continuously, down to $\bar{\chi }≃0$ as Δr reaches $w_{0}≃0.35\;\mu m$, and below 0 to a minimum of $\bar{\chi }≃-0.2$ for $\Delta r≃2w_{0}$ (Figure 5a). For larger separations $\bar{\chi }≃$ goes back to 0. In addition, the distribution of values for χ broadens when Δr increases. A decrease in molecular brightness (corresponding to an increase in photon noise), also results in a broadening of the peak, but not in a shift of the peak position, as expected since the systematic effect of photon noise is corrected for when calculating χ (data not shown). Thus, the average value of χ reflects the distance between the particles when it is below the diffraction limit. Similar conclusions are obtained from the simulation of mobile particles (data not shown).

As the issue of accidental co-localization becomes more preponderant at high surface concentration of particles, we also performed simulations where the concentration of the particles in the second channel was increased up to 200 particles per image (2 particles per $\mu m^{2}$). When particles in both channels are stationary (Figure 5b), the fraction of particles for which χ>0.3 increases up to ≃14%, as more particles in the second channel accidentally co-localize with detected particles in the first channel. Interestingly, this is accompanied by a comparable increase in the fraction of particles with χ<−0.3, as some of the particles in the second channel happen to be within a distance $w_{0}$ to $2w_{0}$ of the detected particles. Similar results are obtained when the particles in the second channel are mobile, except that the frequency of accidental co-localizations is slightly lower (SI Figure S4). Thus, accidental co-localizations, when the detected particles are immobile (and regardless of whether the particles in the second channel are mobile or immobile), have a characteristic signature which is different from that of true colocalization events (which never result in χ<−0.3).

The situation is different when the detected particles are mobile (Figure 5c), since in that case accidental co-localizations (i.e., events with high χ) are much less likely to occur, even at high surface concentration of particles in the second channel. This is both because events have a smaller footprint and because each mobile particle has a unique trajectory and therefore a unique shape in the confocal image, which is unlikely to be accidentally matched by that of another particle. Calculating the particle cross-correlation coefficient is therefore especially interesting in this case. In our experimental conditions (mobile particle concentration of 0.15 particles/$\mu m^{2}$), we expect accidental co-localizations to occur for only 4% of events (if choosing χ=0.3 as the detection threshold) or 1.7% of events (if choosing χ=0.5 as the detection threshold).

#### 2.3.2. Co-Localization of tBid and Bax

**Figure 6:**
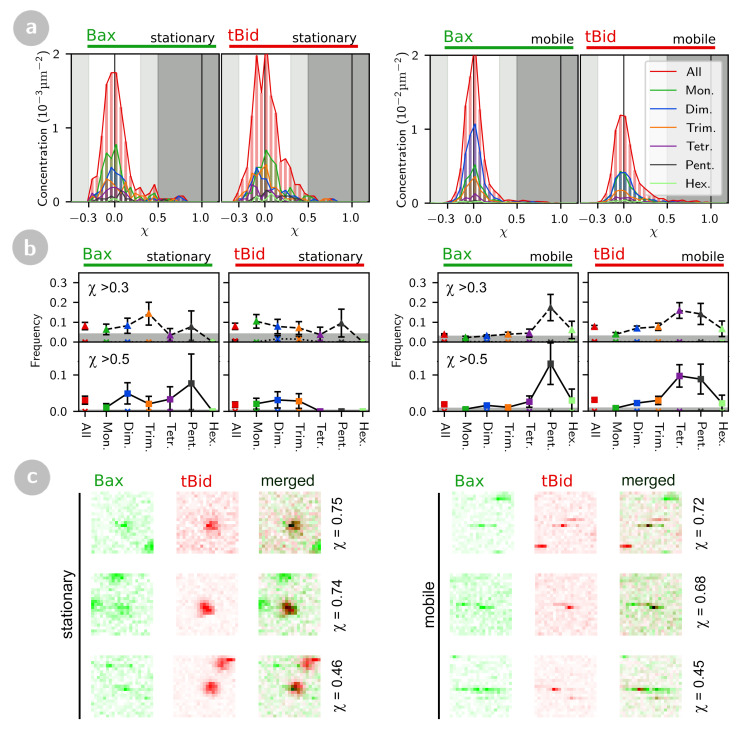
Particle cross-correlation coefficients measured for stationary and mobile tBid and Bax oligomers. (**a**) Cross-correlation coefficient distributions for all oligomers. The light grey areas correspond to slight cross- or anti-correlation (0.3<χ<0.5 or −0.5<χ<−0.3) and the dark grey area highlights strong cross-correlation (χ>0.5). (**b**) Fraction of correlated particles (χ>0.3, upper panels, triangles and dashed lines) and strongly correlated particles (χ>0.5, lower panels, squares and continuous lines) in each oligomer class. The highlighted areas show the absolute maximum amount of accidental positive correlations to be expected given a particle concentration similar to that observed in experiments (0.05 particles/$\mu m^{2}$ for stationary particles and 0.15 particles/$\mu m^{2}$ for mobile particles), as obtained from the simulations shown in Figure 5b,c. In each panel, the fraction of detected anti-correlated particles (χ<−0.3 or χ<−0.5) are also shown (X symbols and dotted lines). (**c**) Representative examples of detected particle pairs with either slight or strong correlation. The shown particle cross-correlation coefficient is the one calculated for the green particle.

The result of the particle cross-correlation analysis applied to the set of confocal images acquired for SLB incubated with tBid-Alexa647 and Bax-HiLyte488 is shown in Figure 6, where the distributions shown are aggregated data for all the Bax concentrations studied. All these distributions (whether the particles are detected in the tBid or the Bax channel, and whether particles are stationary or mobile) show a dominant narrow peak exactly centred at χ=0 (corresponding to a large population of non-interacting particles), a second smaller peak centred at χ = 0.3–0.5 (corresponding to a population of particles with significant cross-correlation with a particle in the other channel), and a few rare events with χ>0.5 (corresponding to particles positively bound to a particle in the other channel). Almost no events with clear anti-correlation (χ<−0.3) are detected.

Concentrating first on stationary particles (Figure 6, two left panels), we see that the number of positively correlated events is above the expected maximum number of false positives (lower panels), both when considering all events together, and when separating them into different stoichiometries. In addition, since these positively correlated events are not accompanied by any negatively correlated events, they do not fit the pattern expected for accidental co-localizations. We therefore considered all stationary events with χ>0.3 as corresponding to tBid-Bax complexes.

In the case of mobile tBid oligomers, the frequency of positively correlated events (whether they are defined as χ>0.3 or χ>0.5) is significantly larger than what would be expected for accidental co-localizations (Figure 6, right panels), and becomes very substantial for larger oligomers. The case is not as clear cut for mobile Bax oligomers, as the levels of detected positively correlated events are very close to the maximum expected level for accidental co-localizations, except for larger Bax oligomers where an above-background positive correlation is clearly seen. We note that when separating the data according to the Bax concentration used for incubation, no obvious trend was observed (SI Figure S5). For mobile particles, we conservatively estimated the number of tBid-Bax complexes by subtracting the maximum number of accidental co-localizations from the number of mobile events with χ>0.3.

#### 2.3.3. Dissociation Constant of tBid-Bax Complexes

The two-dimensional dissociation constant for tBid and Bax ($2D-K_{D}$), was obtained using either the tBid or the Bax channel to detect complexes, and for particles with different mobilities or stoichiometries. The $2D-K_{D}$ was calculated from the observed surface concentrations of non-interacting particles ($c_{\mathrm{Bax}}$ and $c_{\mathrm{tBid}}$ with $\chi <\chi_{\mathrm{tresh}}$) and of interacting complexes ($c_{\mathrm{Bax}\text{-}\mathrm{tBid}}$, calculated from the number of events with χ>0.3 in the considered channel, and in the case of mobile tBid subtracting the maximum number of accidental co-localizations predicted from simulations), using Equation (7). Dissociation constants have the dimension of a concentration, thus the $2D-K_{D}$ has the dimension of a surface concentration. When considering all membrane species of tBid and Bax (mobile and stationary, regardless of stoichiometry and initial Bax concentration), we find an apparent dissociation constant $2D-K_{D}=1.1\;\mu m^{-2}$. However, separating the data into mobile particles (using the tBid channel to detect complexes) and stationary particles (using either the tBid or the Bax channel to detect complexes) show that the nature of that equilibrium changes significantly when the proteins change conformation: the complex is much more stable for stationary (membrane-inserted) proteins ($2D-K_{D,\mathrm{stationary}}=0.1\;\mu m^{-2}$) than for mobile (loosely-bound) proteins ($2D-K_{D,\mathrm{mobile}}=1.6\;\mu m^{-2}$).

**Figure 7:**
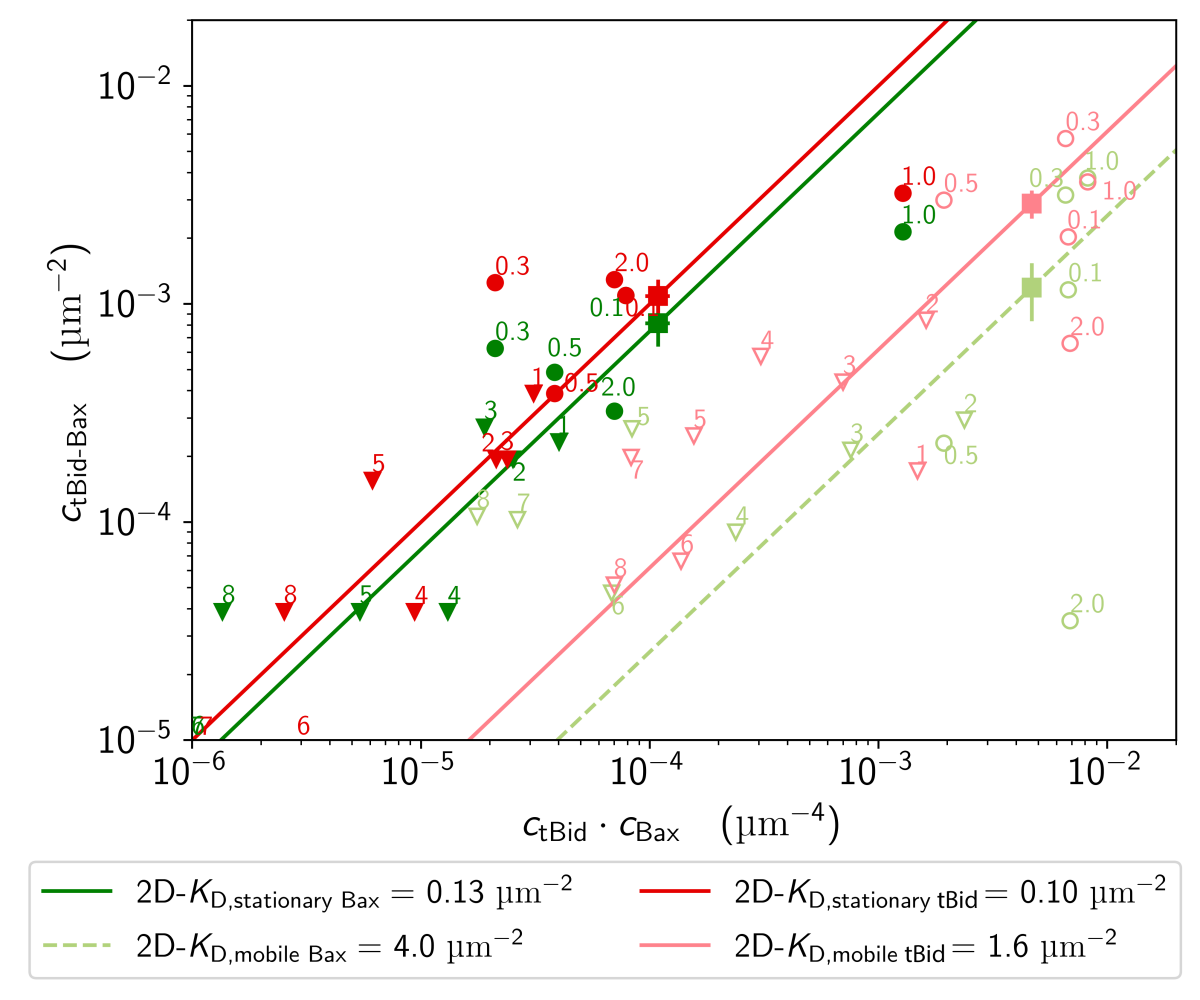
Surface concentration of the tBid-Bax complexes (defined as particles with $\chi_{\mathrm{thresh}}>0.3$) detected either in the Bax channel (green symbols) or the tBid channel red symbols) as a function of the product of the reactants. Data are shown both for stationary particles (dark red and dark green symbols) or mobile particles (light red and light green symbols). Each circle represents the aggregated data for a specific Bax concentration used for incubation (indicated on the figure, in nM), while each triangle represent the aggregated data for a specific oligomer size (also indicated on the figure). Squares represent the value obtained for the whole data set. The solid lines are fit of the data with Equation (7) for stationary proteins on the one hand and mobile proteins on the other hand, assuming a constant $2D-K_{D}$ (on this log-log plot the slope of these lines is 1 and their vertical offset, $\log[2D-K_{D}^{-1}]$ is linked to the inverse of the dissociation constant).

In contrast, we see no significant change in the dissociation constant when considering samples incubated with different Bax concentrations, or populations of either tBid or Bax with different apparent stoichiometries. This is illustrated in Figure 7, which shows the concentration of protein complexes, $c_{\mathrm{Bax}\text{-}\mathrm{tBid}}$, as a function of the product $c_{\mathrm{Bax}}\;c_{\mathrm{tBid}}$, for all these different populations. Populations with the same $2D-K_{D}$ should lay on the same line of slope 1. All subpopulations of mobile particles (regardless of the initial Bax concentration or particle stoichiometry) more or less fall on a line corresponding to $2D-K_{D,m}≃1.6\;\mu m^{-2}$, while all subpopulations of stationary particles fall on a $2D-K_{D,s}≃0.1\;\mu m^{-2}$ line. Thus, the main determinant of the $2D-K_{D}$ is the conformation of the protein and its degree of insertion in the membrane.

## 3. Discussion

**Figure 8:**
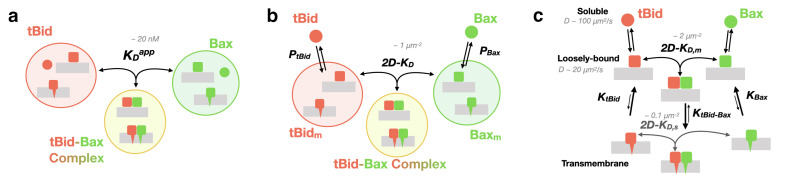
Three different levels of models for the tBid-Bax interaction. (**a**) In the simplest model, all the forms of tBid, Bax and the tBid-Bax complex are each considered as a separate conformational ensemble. The interaction between the tBid and Bax ensembles is described by a simple apparent dissociation constant, $K_{D}^{app}$. (**b**) An improved version of the simple model in (a) considers the solution and membrane forms of each protein as separate conformational ensembles (the tBid-Bax complexes exist only as membrane-associated complexes). At equilibrium the system is described with two partition coefficients, $P_{tBid}$, $P_{Bax}$, characterizing the equilibrium (retro-translocation) between the solution and membrane forms of each protein, and a two-dimensional dissociation constant, $2D-K_{D}$. (**c**) A more accurate model, where the membrane forms of each protein are separated into two different conformational ensembles based on their degree of insertion in the membrane (but regardless of stoichiometry), is necessary to explain the results of our single particle experiments. The equilibrium of this system is described by four independent partition coefficients, and two two-dimensional dissociation constants, $2D-K_{D,m}$ for the mobile loosely-bound proteins and $2D-K_{D,s}$ for the stationary deeply inserted proteins.

In the present work, by combining the ideas of single particle detection (which let us identify and characterize specific protein complexes) and image cross-correlation spectroscopy (which let us identify real protein–protein interactions) we were able to directly measure the two-dimensional dissociation coefficient of the membrane-associated tBid-Bax complex. This was achieved in spite of the extremely low fraction of interacting proteins found at the low surface concentrations (below the $2D-K_{D}$) necessary to reach single particle detection conditions. The reconstituted system we have used differs from in vivo cellular systems in several ways, amongst which: the lipid membrane in our reconstituted system is planar, supported by a solid substrate and devoid of proteins other than tBid and Bax; we chose to use human Bax and murine tBid, for direct comparison with previous studies that looked at quantifying the tBid/Bax interaction (and had shown these two proteins had a direct interaction and that together they were able to permeabilize membranes [10,34,52,53,54]); the purified tBid and Bax proteins added to the system have each been labelled with a small organic fluorophore, and Bax has been subjected to three point mutations; for convenience the experiments were performed at room temperature rather than physiological temperature. Because of these differences, the dissociation constants we report here can be expected to be slightly different from those encountered in vivo in human cells. However, our measurements: (*i*) bring a proof-of-principle that two-dimensional dissociation constants for membrane proteins can be measured from confocal images using the concept of particle cross-correlation coefficient; and (*ii*) allow comparing the dissociation constants for several membrane conformations of tBid and Bax. Indeed, our most important finding is that the stability of the tBid/Bax complex depends not only on whether tBid and Bax are in a soluble form or in a membrane form, but also on their membrane conformation, a refinement of our current understanding of Bax activation which is summarized in Figure 8.

If one does not distinguish between the soluble and membrane forms of the proteins, the equilibrium of the system can simply be described by an apparent dissociation constant (Figure 8a). This dissociation constant was previously measured, using ensemble fluorescence resonance energy transfer, and found to be $K_{D}^{app}=20$ nM in a liposome solution with total lipid concentration [L]=125 μM [10]. However, this apparent dissociation constant is insufficient to describe the tBid/Bax system. First, tBid and Bax hardly interact in their soluble form [10,26]. This is true of many other pairs of Bcl-2 family proteins and the tenet of the “embedded together” model which emphasizes the importance of the membrane in enabling interactions between Bcl-2 family proteins [16,55]. Second, most Bcl-2 family proteins are in a dynamic equilibrium between their soluble and membrane forms, a phenomenon known as “retro-translocation” [22,23,24,56]. These considerations have led to models such as the one illustrated in Figure 8b, where the proteins exists both in soluble and membrane forms, and where regulation of the equilibrium between the two forms of Bax becomes another way of controlling its activity [2,5,57]. The equilibrium of the system is then characterized by two partition coefficients ($P_{tBid}$ and $P_{Bax}$) describing the equilibrium between the soluble form and membrane form of tBid and Bax, and one two-dimensional dissociation constant for the membrane tBid/Bax complex ($2D-K_{D}$). The value of this dissociation constant can be estimated from the measured value of $K_{D}^{app}$, since comparing these two models shows that $2D-K_{D}\approx K_{D}^{\mathrm{app}}\times d\left[L\right]v_{L}P_{tBid}P_{Bax}$, where d=4 nm is the membrane thickness and $v_{L}=7.6\times 10^{-4}$ $L\;{\mathrm{mol}}^{-1}$ is the molar volume of the lipids (see supplemental material and Figure S1 for a derivation and discussion of this relationship). Considering that $P_{tBid}=7000$ [29] and assuming $P_{Bax}=3500$, leads to the order of magnitude estimate $2D-K_{D}∼0.1\;\mu m^{-2}$.

Our single-particle detection confocal experiments, however, show that the model shown in Figure 8b is oversimplified, by confirming the multiplicity and complexity of the tBid and Bax membrane forms glimpsed in previous studies, with both proteins in equilibrium between at least two different types of membrane conformations. In the first ensemble of conformations, the proteins have a very low mobility (D=0 to $0.5\;\mu m^{2}\;s^{-1}$ in the SLB), signalling that they must be inserted in the bilayer with one or more helices going all the way through. The existence of transmembrane conformations linked to membrane permeabilization was suggested for both tBid and Bax by single residues fluorescence labelling experiments [43,58], and for tBid by single particle tracking experiments [29]. Solving the membrane structure of these proteins has proven challenging, yet models for transmembrane forms of Bax have been proposed based on x-ray, electron paramagnetic resonance and fluorescence experiments [58,59,60,61] or simulations [62]. Models also exist for tBid [43,63,64], although the consensus in this case is that tBid is only partially inserted into the membrane. In contrast to these membrane-embedded forms, the second detected membrane conformational ensemble is characterized by a very high mobility (D≃10 to $20\;\mu m^{2}\;s^{-1}$), considerably larger than that of the lipids ($D_{lipid}≃2$ to $3\;\mu m^{2}\;s^{-1}$ in mitochondria-like SLB [29,46]). This second fraction thus corresponds to loosely bound proteins, probably associated to the membrane via electrostatic interactions, and probably retaining a structure close to that of the soluble form (as suggested for Bax by simulations [62] and small-angle scattering experiments [65]). This population has been observed before in the same system for tBid using single-point FCS, and its average diffusion coefficient estimated to be $D=22\;\mu m^{2}\;s^{-1}$ [29]. The loosely-bound form, appearing as streaks in our confocal images, is too fast to be detected in camera-based single-particle tracking experiments, thus its existence has not often been acknowledged. Despite its elusiveness in most experiments, this form is probably crucial in vivo, both by allowing a dynamic equilibrium with the soluble form of the proteins (retro-translocation) and by allowing the protein to perform a quick two-dimensional search for binding partners on the membrane (electrostatic scanning [66]).

Our results show that there is one dissociation constant associated with the mobile species superficially bound to the membrane, $2D-K_{D,m}≃1.6\;\mu m^{-2}$, and a second significantly lower one associated with the stationary transmembrane species, $2D-K_{D,s}≃0.1\;\mu m^{-2}$. This leads us to propose the model shown in Figure 8c, where, for each protein, the membrane conformational ensemble is explicitly separated into loosely-bound (mobile) and transmembrane (stationary) species, and within each of these ensembles the tBid and Bax binding equilibrium is characterized by a different dissociation constant. It is striking that, whereas the dissociation constant we measure for the superficially bound tBid/Bax complex is comparable to that measured in vivo for membrane receptor dimerization [31,67], the dissociation constant measured for the deeply inserted tBid/Bax complex is one order of magnitude lower, signalling a much tighter interaction. The introduction of a loosely bound state for tBid and Bax with a detectable but weak affinity for each other is consistent with the notion that interactions between Bcl-2 family proteins are tighter when the proteins are interacting with the membrane, with the added intricacy that the affinity between the two proteins progressively increases as they get inserted deeper into the membrane. This model is also consistent with the finding that Bax oligomers can form before Bax membrane insertion [68].

The model shown in Figure 8 is still likely a simplification, since loosely-bound transmembrane species might be able to directly interact, and stoichiometry of complexes are not taken into account. It also says nothing of the actual pore formation process which comes after Bax activation due to the formation of large Bax oligomers. Large complexes were detected in our system, both for tBid and Bax, and a fraction of these large complexes were mixed complexes that contained both Bax and tBid proteins (an observation that is somewhat reminiscent of the recent finding that oligomers of Bcl-XL can bind both tBid and Bad at the same time [69]). This is surprising, because the tBid/Bax interaction is often thought of as a hit-and-run interaction, where the tBid/Bax complex dissociates before the activated Bax can bind other Bax molecules (allowing tBid to activate more Bax)—instead, it seems that either tBid and Bax can remain associated as the Bax oligomers grow or that tBid is able to re-bind to already active oligomeric Bax. The capacity of tBid to participate in large oligomeric complexes, usually thought to be specific to the multidomain effector proteins (Bax and Bak), might be related to the fact that it is structurally related to Bax [70,71]. It might thus not be representative of the behavior of other BH3-only activator proteins, which do not share this structural homology.

Our work also informs on the dynamics of the tBid/Bax interaction. A stable and short range interaction (on the timescale of the confocal imaging performed here, i.e., the time to image a single particle, 0.1 to 0.3 s in the conditions of our experiments) would result in a particle cross-correlation coefficient close to χ≃1 (as shown by our simulations, see Figure 5a). Instead, the experimental values of χ we measured for interacting particles were lower, around 0.3 to 0.8. This is not an artefact due to imperfect channel overlap, as values χ≃1 are obtained for doubly-labelled liposomes [33]. One intriguing possibility would be that instead of being the result of a direct molecular interaction between tBid and Bax, the complexes that we detect are in fact accumulation of proteins in small ≃100 nm lipid domains. This would explain the very varied stoichiometries observed for both proteins separately as well as for the mixed tBid/Bax complexes, but it would not be totally consistent with the fact that direct contact can be detected between both proteins using FRAP [10]. Perhaps a more likely possibility is that this imperfect correlation is due to the transient nature of the tBid/Bax interaction, which is known to be dynamic [10] and was suggested to be short lived [26]. A tBid/Bax interaction with a lifetime on the order of 0.1 s (similar to the time necessary to record the few lines in the confocal image in which the complex appears) would result in a value of χ that is positive but less than 1, as we observed.

Recent protein knockout experiments have shown that Bax can be activated without the help of any of the eight canonical BH3-only proteins [72,73]. This calls into question the biological relevance of the direct activation model, which is centred around the idea that BH3-only activators (such as tBid and Bim) activate effector proteins (Bax and Bak) through direct physical interaction [19]. It is true that ever since Bid was first identified in a screen of proteins binding to Bax [34], a direct interaction between tBid and Bax has remained hard to detect in live cells (which is maybe not so surprising since this interaction is so strongly correlated with cell death). On the other hand, as shown in multiple reconstituted lipid systems, there is no question that tBid and Bax interact as long as a lipid membrane is present, and that tBid triggers the membrane localization and the pore-forming activity of Bax [7,10]. Even so, as far as we know, our experiments represent the first time the tBid/Bax interaction could be observed at the single particle level (Figure 6).

Following this challenging of the direct activation model, it has been proposed that the mitochondrial membrane (and not BH3-only activators such as tBid) played the role of Bax activator [19]. This proposal is consistent with observations that a cardiolipin-rich membrane can lead to Bax auto-activation [74]. However, cardiolipin appears in the outer mitochondrial membrane only after apoptosis is triggered, so in cells this activation mechanism might be used for amplification rather than initiation. In any case, in order to be activated by or with the help of the membrane, Bax needs to be brought and held at the membrane. This might be precisely the role of activator proteins such as tBid and Bim, which have strong affinities for the membrane and can associate with it quickly and almost irreversibly. Indeed what stands out in the tBid/Bax interaction model inspired by our experiments (Figure 8c) is that the interaction between tBid and Bax seems designed to pull Bax down and anchors it in the membrane, maybe just long enough for Bax to become activated by the membrane, and maybe greatly accelerating a process which may happen in the absence of tBid, but would be much slower. This formalizes an already existing narrative for the tBid/Bax interaction, which is that tBid “recruits” Bax to the membrane, and can shift the equilibrium between the soluble and membrane forms of Bax [22,23,24,75].

Eventually, a strong test of the direct interaction model will be to directly compare the affinity of tBid for Bax to that of tBid for Bcl-XL. Values are available for the $K_{D}$ of tBid/Bax (20 nM) [10] and tBid/Bcl-XL (1 nM) [76]) in the presence of membranes. However, these values cannot be compared, because they have been measured in different reconstituted systems (liposomes vs. mitochondria) and without knowing exactly how much of the proteins was at the membrane. Considering only the membrane fraction of the proteins and measuring the $2D-K_{D}$ like we have done here resolves this issue. If indeed the direct interaction model is irrelevant for tBid, then it should be possible to show that the $2D-K_{D}$ for the Bcl-XL/Bid interaction is much smaller than that for the tBid/Bax interaction. In the future, the method employed here can also be applied to compare the $2D-K_{D}$ of different tBid and Bax mutants (e.g., using mutations in the BH3 motif of tBid in order to modify the affinity of tBid for Bax, Bcl-2 and Bcl-XL [34,77]).

## 4. Materials and Methods

### 4.1. Protein Purification and Labelling

#### 4.1.1. tBid

Full-length murine Bid with a N-terminal His-tag and a cysteine to serine mutation at position 30 (leaving a single cysteine residue at position 126, and thus referred to as Bid 126C), was purified as previously described [10,28,43,76]. Briefly, a plasmid encoding the Bid 126C construct was transformed into *E. coli* BL21 arabinose inducible cells by electroporation. Single colonie large scale 5 L cultures in lysogeny broth (LB) were induced with 0.2% arabinose for 3 h at 37 °C. Cells were then spun down at 5000 g for 30 min at 4 °C and the pellet was resuspended in Bid lysis buffer: 10 mM HEPES pH 7, 100 mM NaCl, 10 mM imidazole, 1 mM PMSF, 10 μg m$L^{-1}$ DNase and 1× Halt Proteinase Inhibitor Cocktail (Thermo Fischer, Waltham, MA, USA). Cells were then vortexed, passed through a 16 gauge needle and lysed by at least 2 passes through a French Press. Cell debris were removed by centrifugation at 15,000 *g* for 30 min at 4 °C. The supernatant was incubated with 1.5 mL Ni-NTA agarose resin (Qiagen, Hilden, Germany) for 1.5 h at 4 °C with mixing. The slurry was added to a 20 mL column (Bio-Rad, Hercules, CA, USA) that retained the resin, after which the column was washed with 50 mL Bid wash buffer: 10 mM HEPES pH 7, 300 mM NaCl, 1% (*w*/*v*) CHAPS (Bioshop Canada, Burlington, ON, Canada) and 10 mM imidazole. The protein was eluted in Bid elution buffer (10 mM HEPES pH 7, 100 mM NaCl, 0.1% (*w*/*v*) CHAPS, 200 mM imidazole and 10% glycerol).

The purified Bid 126C was then fluorescently labelled with Alexa647 (Molecular Probes, now Thermo Fisher), which formed a covalent bond via maleimide chemistry to the cysteine residue at position 126 [43]. Briefly, proteins in Bid storage buffer (10 mM HEPES pH 7, 100 mM NaCl, 0.2 mM EDTA, 10% glycerol) supplemented with 0.5% *w*/*v* CHAPS were incubated with a 10-fold molar excess of dye for 2 h at room temperature and in the dark while under constant rotation. Unreacted free dye was then first quenched by addition of 1 mM DTT, then removed by gel filtration in a G-25 fine Sephadex column (GE Healthcare, Chicago, IL, USA). The labelling efficiency was determined from absorption measurements that gave both protein and dye concentrations, and found to be 70% in this case.

The fluorescently labelled Bid was then cleaved with Caspase-8 to obtain cleaved Bid (cBid), as described in Ref. [43]. The fluorescent label (at residue 126) is on the larger C-terminal fragment of the protein, referred to as truncated Bid (tBid). Since both the His-tag and the C30S mutation are on the small N-terminal fragment of Bid, the only difference between purified tBid-Alexa47 and wild type tBid is the presence of the fluorescent Alexa647 tag.

#### 4.1.2. Bax

Full-length human Bax, with the two endogenous cysteine residues (at positions 62 and 126) replaced with alanine, and with an additional leucine to cysteine mutation at position 47 (Bax 47C) was purified as previously described [10,58,76,78]. Briefly, a plasmid encoding the Bax 47C construct fused to a C-terminal chitin binding domain followed by a self-cleaving intein sequence (intein-chitin binding domain or CBD, New England Biolabs, Ipswich, MA, USA) was transformed into *E. coli* BL21 arabinose inducible cells by electroporation. Single colonies were selected for large scale 5 L cultures in lysogeny broth (LB), and induced with 0.2% arabinose for 5 h at 30 °C. Cells were then spun down at 5000 g for 30 min at 4 °C. The pellet was resuspended in Bax lysis buffer: 10 mM HEPES pH 7, 100 mM NaCl, 0.2% (*w*/*v*) CHAPS, 1 mM PMSF, 10 μg m$L^{-1}$ DNase and 1× Halt Proteinase Inhibitor Cocktail (Thermo Fischer). Cells were then vortexed, passed through a 16 gauge needle and lysed by at least 2 passes through a French Press. Cell debris were removed by centrifugation at 15,000× *g* for 30 min at 4 °C. The supernatant was incubated with 2 mL chitin resin (New England Biolabs) for 1.5 h at 4 °C with mixing. The slurry was added to a 20 mL column (Bio-Rad) that retained the resin, after which the column was washed with 50 mL Bax wash buffer (10 mM HEPES pH 7, 500 mM NaCl, 0.5% (*w*/*v*) CHAPS), followed by flushing with 60 mL Bax cleavage buffer (10 mM HEPES pH 7, 200 mM NaCl, 0.2 mM EDTA, 0.1% (*w*/*v*) CHAPS, 100 mM hydroxylamine). The column was then incubated in Bax cleavage buffer for 36 to 48 h at 4 °C, after which the protein was eluted in Bax cleavage buffer. Hydroxylamine was removed by dialysis in Bax storage buffer (10 mM HEPES pH 7, 200 mM NaCl, 0.2 mM EDTA, 10% (*w*/*v*) glycerol).

Purified Bax was labelled with the small neutral dye HyLite488 (Anaspec, Fremont, CA, USA) through maleimide chemistry, as described above for Bid, but at pH 7.5 in presence of a 2-fold molar excess of TCEP to promote reduction of the thiol group, and the labelling reaction was allowed to proceed for a total of 6 h. Free dye was removed and labelling efficiency was assessed as for Bid. The labelling efficiency in that case was found to be 81%. We note that labelling was also attempted for several different dyes (Alexa488, Atto488, Atto495) and one other position (residue 126), but these resulted in very low labelling efficiencies, as did the protocol without TCEP. To confirm that labeling occurred only at the cysteine residue, control experiments were done with a cysteine-less mutant of Bax and wild-type Bax. As expected, labelling was very low in the first case (11%) and about double that of Bax 47C in the second case (155%).

#### 4.1.3. Purified Protein Activity

Since the purified and labeled proteins differ from their wild-type equivalent due to the presence of a fluorescence tag (and, in the case of Bax, due to the presence of three point mutations) it was important to test their activity. This was done for each batch of purified and fluorescently labelled protein using a standard ANTS release assay [78]. The pore-forming activity of cBid-Alexa647 (assessed in conjunction with purified unlabeled wild-type Bax), was found to be slightly (10 to 20%) higher than that of its wild-type equivalent. In contrast, the pore-forming activity of Bax-HyLite488 (assessed in conjunction with purified unlabeled cBid) was found to be lower (30 to 40%) than that of its wild-type equivalent. When using both labelled proteins together in the assay, the pore-forming activity was reduced by only 20 to 30% when compared to the corresponding wild-type proteins, which was judged acceptable.

### 4.2. Mitochondria-Like Supported Lipid Bilayers

Mitochondria-like solid-supported lipid bilayers were prepared by liposome fusion [29]. The lipid composition was chosen to represent that of mitochondrial membranes in yeast and vertebrates [79,80], with phosphatidylcholine (PC), phosphatidylethanolamine (PE), phosphatidylinositol (PI), phosphatidylserine (PS) and cardiolipin (CL) in a molar ratio of 48:28:10:10:4. All lipids were acquired from Avanti Polar Lipids (Alabaster, AL, USA) as either egg extracts (PC, PE), liver extracts (PI) or monounsaturated synthetic lipids ((18:1)${}_{2}$ PS, (18:1)${}_{4}$ CL). The different lipids, dissolved in chloroform, were mixed in the right proportion to reach a total lipid mass of 1 mg, after which the chloroform was allowed to evaporate first under a stream of argon and later in a vacuum at 25 inHg for at least 2 h. The lipid film was rehydrated with 1 ml of an assay buffer solution (10 mM HEPES pH 7, 200 mM KCl, 1 mM MgCl${}_{2}$, and 0.2 mM EDTA), followed by 10 freeze-thaw cycles to obtain unilamellar liposomes, and extrusion through a 100 nm pore filter. The liposome solution was then injected into a 400 μL perfusion chamber (Bioptechs FCS2, Butler, PA, USA), fitted with a mica-coated glass coverslip, the manufacture of which is important both for proper SLB formation and confocal image quality. In order to ensure that the total thickness of the coverslip was less than 220 μm (the maximum thickness acceptable given the objective used for imaging), glass coverslips with thickness 170 μm were used, and the mica (25 mm diameter circular sheet, V-1 grade, purchased from SPI Supplies, West Chester, PA, USA) was first cleaved to a thickness of 6 to 12 μm (as determined from its weight). The mica sheet was gently pressed down onto the glass coverslip coated with an optical adhesive (Norland Products, Cranbury, NJ, USA) pre-heated to a temperature of 50 °C, and the assembly was exposed to UV illumination to fix the glue. Just before perfusion chamber assembly and lipid solution injection, the top layers of the mica were removed using clear packing tape to expose a clean mica surface. The sample was incubated at 37 °C for 1 h in order to give time to the liposome to fuse onto the mica surface and form a single SLB. The lipid membrane was washed by slow injection of assay buffer in the chamber, after which a 0.2 nM solution of cBid-Alexa647 in assay buffer was injected, followed by a 15 min incubation period at 37 °C. A Bax-HyLite488 assay buffer solution (with Bax concentration varying between 0.1 and 2 nM) was then injected in the chamber, again followed by an incubation period (2 h at 37 °C).

### 4.3. Confocal Imaging

Fluorescent proteins associated with the SLB were imaged using a dual-colodr confocal fluorescence fluctuation microscope (Insight, Evotec Technologies, Hamburg, Germany, now Perkin-Elmer, Waltham, MA, USA). Excitation was provided simultaneously at $\lambda_{\mathrm{ex}}=488\mathrm{nm}$ and $\lambda_{\mathrm{ex}}=635\mathrm{nm}$ by two lasers (Sapphire 488-20 CDRH and Radius 635-25, both from Coherent, Santa Clara, CA, USA), whose beams were combined into a single optical fiber, before being focused into the sample using a water immersion objective (U-Apo, 40×, 1.15 NA, Olympus, Tokyo, Japan). The fluorescence signal was refocused through a 40 μm confocal pinhole, separated into green and red detection channels by a dichroic mirror and detected by two avalanche photodiodes (SPCM-CD3017, Perkin-Elmer). Before each experiment, the confocal pinhole was aligned and the observation volume was calibrated in both channels by performing a FCS experiment using nanomolar solutions of Alexa488 (diffusion coefficient $D=390\;\mu m^{2}\;s^{-1}$ at 20 °C [81], $\lambda_{\mathrm{em}}=525\mathrm{nm}$) and Alexa647 (diffusion coefficient $D=290\;\mu m^{2}\;s^{-1}$ at 20 °C [82], $\lambda_{\mathrm{em}}=665\mathrm{nm}$), both purchased from Molecular Probes (now Thermo Fisher). Calibration experiments were performed in a sample chamber prepared with a microscope coverslip coated with mica, exactly as the SLB sample to be imaged. Typically, the $1/e^{2}$ radius of the detection volume was estimated to be $w_{g}=320\mathrm{nm}$ in the green detection channel and $w_{r}=370\mathrm{nm}$ in the red detection channel, a bit larger than the expected values in ideal conditions $w_{g}=0.51\left(\lambda_{\mathrm{ex}}+\lambda_{\mathrm{em}}\right)/2/{\left(2\ln2\right)}^{1/2}/\mathrm{NA}=190\mathrm{nm}$ and $w_{r}=245\mathrm{nm}$. A second calibration step was then carried out using nanomolar solutions of Bax-HyLite488 and cBid-Alexa647, also through a mica-coated coverslip, to determine their molecular brightness. At the 20 μW excitation laser power used in each channel for these experiments, the molecular brightness of Bax-HyLite488 was found to be $B_{g}=$ 6(1) kHz and that of cBid-Alexa647 to be $B_{r}=$ 11(2) kHz. Finally, dual-color z-stacks of 10 μm by 10 μm (100×100 pixels) images were acquired with an oversampling pixel size d=100 nm and a pixel dwell time δ=1 ms for multiple regions of interest in each sample. Typically, each stack contained 5 or 10 pairs of images, spaced by 0.5 or 1 μm, centred around the focal plane. For each stack, the pair of images with the highest maximum intensity was considered to correspond to the images for which the membrane plane was in focus, and selected for single particle analysis.

### 4.4. Simulations

To validate the particle detection and classification scheme used on confocal images, a program was written in Python (using the NumPy and Pandas libraries) to simulate particle diffusion in a plane and generate confocal images of this plane (with dimensions $n_{x}\times n_{y}=100\times 100\;\mathrm{pxl}$, pixel size d=100 nm and pixel dwell time δ=1 ms). To emulate the behaviour of particles which may move during the recording of a pixel intensity, the time step for the simulation τ was chosen to be a fraction of the exposure time, τ=δ/m, with m=10. Initial positions for *N* different particles, ${\vec{r}}_{0}$, were randomly selected inside the image area. Tracks for mobile particles were simulated with the constant diffusion coefficient *D*, by generating at each time step *n* a displacement $\delta \vec{r}\left(n\right)=\left(\delta x,\delta y\right)$ for each particle, whose components were drawn from a Gaussian distribution:

(1) $$\Psi \left[\delta x\right]=\frac{1}{\sqrt{4\pi D\tau }}\exp\left\{-\frac{\delta x^{2}}{4D\tau }\right\}.$$

With the knowledge of all particle positions at all times (${\vec{r}}_{p}\left(t\right)$), the confocal image was generated on a pixel-by-pixel basis, were the intensity at pixel (i,j) is recorded starting at time $t_{ij}=\left(n_{x}j+i\right)\delta$. Assuming a Gaussian beam profile with $1/e^{2}$ radius $w_{0}$, a particle found at a distance $\Delta \vec{r}$ from the center of the pixel contributes on average the following number of photons during the time interval τ:

(2) $$g\left[\Delta \vec{r}\right]=B\tau \exp\left\{-\frac{2{\left(\Delta \vec{r}\right)}^{2}}{w_{0}^{2}}\right\},$$

where *B* is the molecular brightness of the particle. For the simulations we used $w_{0}=0.3\;\mu m$ and B=5 to 20 kHz. The average pixel photon intensity recorded at pixel (i,j) between *t* and t+τ can then be calculated by summing over all particles and over the whole dwell time:

(3) $$I\left(i,j\right)=\sum_{n=0}^{m-1}\sum_{p=1}^{N}g\left[{\vec{r}}_{ij}-{\vec{r}}_{p}\left(t_{ij}+n\tau \right)\right]+i_{B}\delta ,$$

where the constant background intensity of $i_{B}=1.3\mathrm{kHz}$ was added at each pixel, to match the typical background intensity measured in the experimental confocal images. Finally, photon noise was added to the image, by replacing the average photon count calculated using Equation (3) by a photon count drawn from a Poisson distribution with the average pixel intensity as the expectation value.

The Python code used to simulate confocal images is available in the publicly accessible repository: https://github.com/cecilefradin/BidBax_Simulation_and_Analysis (accessed on 30 July 2021).

### 4.5. Image Analysis

The confocal images were analyzed using an in-house algorithm, written as a Java plugin for ImageJ (https://github.com/cecilefradin/BidBax_Simulation_and_Analysis, accessed on 30 July 2021). This algorithm detects single particles in one channel, determines their size, shape and fluorescence intensity, then calculate for each one a single particle cross-correlation coefficient, which allows establishing whether it is bound to another particle in the second channel. The intensity distribution for a population of particles inform about the distribution of stoichiometries in that population, while the surface concentrations of interacting and non-interacting particles allow calculating two-dimensional dissociation constants.

#### 4.5.1. Single Particle Detection

Particle detection and identification was done as detailed in [29]. Images were first searched for local maxima. Each maximum with intensity above the threshold value $I_{T}=\langle I_{B}\rangle +0.2B$ (with *B* the value of the molecular brightness measured for the solubleprotein by FCS on the same confocal instrument, and $\langle I_{B}\rangle$ initially chosen as the average intensity in a region of 5×5 pixels centred around the pixel with lowest intensity in the image) was considered in turn, starting with the brightest one, and fitted with a two-dimensional Gaussian function:

(4) $$H\left(x,y\right)=I_{p}\;\exp\left\{-\frac{2{\left(x-{\bar{x}}_{p}\right)}^{2}}{w_{x,p}^{2}}\right\}\;\exp\left\{-\frac{2{\left(y-{\bar{y}}_{p}\right)}^{2}}{w_{y,p}^{2}}\right\}+I_{B,p}.$$

This fit returned the particle position $\left({\bar{x}}_{p},{\bar{y}}_{p}\right)$ with sub-pixel precision, the particle fluorescence intensity ($I_{p}$), the $1/e^{2}$ radii of the image of the particle along the *x* and *y* direction ($w_{x,p}$ and $w_{y,p}$) an estimate of the local background intensity ($I_{B,p}$), and a normalized χ-squared value ($\chi_{N,p}$). Once the fit was over, a square region of 0.5 μm× 0.5 μm around the particle was erased from the image and the next most intense remaining local maximum was considered, using an updated threshold value (with $\langle I_{B}\rangle$ now calculated as the average of all the local background intensities estimated from previous fits). Only particles for which the fit was judged acceptable ($\chi_{N,p}<2\;$, $\;I_{p}+I_{B,p}>I_{T}+\sqrt{I_{T}}$) were selected for further analysis.

#### 4.5.2. Single Particle Cross-Correlation Coefficient

To determine whether a particle detected in a given channel interacts with another particle in the other channel, a single particle cross-correlation coefficient was calculated for that particle. First, an area of size 7×7 pixel was delimited around the detected particle. Then the intensities $I_{ch1}$ and $I_{ch2}$ recorded at each pixel within that area for either channels were cross-correlated via:

(5) $$\chi =\frac{\langle \left(I_{ch1}-\langle I_{ch1}\rangle \right)\;\cdot \;\left(I_{ch2}-\langle I_{ch2}\rangle \right)\rangle }{\sqrt{\sigma_{ch1}^{2}-\langle I_{ch1}\rangle }\sqrt{\sigma_{ch2}^{2}-\langle I_{ch2}\rangle }}$$

with

(6) $$\sigma_{ch(k)}^{2}=\langle {\left(I_{ch(k)}-\langle I_{ch(k)}\rangle \right)}^{2}\rangle ,$$

and where averages are taken over the box drawn around the particle. Equation (5) includes a correction for the photon noise, via subtraction of the variance of the Poisson distributed photon noise ($\langle I_{ch(k)}\rangle$) from the total variance ($\sigma_{ch(k)}^{2}$), leaving only the contribution due to the average signal of the particle, as discussed in Friaa and Fradin [33].

#### 4.5.3. Dissociation Constant

A value of the two-dimensional dissociation constant, 2D-$K_{D}$, was obtained by comparing surface concentrations of uncorrelated particles with the channel-averaged surface concentration of the correlated particles. It can be written as:

(7) $$2D-K_{D}=\frac{c_{\mathrm{Bax}}\;c_{\mathrm{tBid}}}{c_{\mathrm{Bax}\text{-}\mathrm{tBid}}},$$

where $c_{\mathrm{Bax}}$ and $c_{\mathrm{tBid}}$ are the respective sums of particles per area with χ<0.3 (no correlated signal in the other channel) and $c_{\mathrm{tBid}\text{-}\mathrm{Bax}}$ is the sum of particles per area with χ>0.3 (correlation of the signals in both channels) corrected for the expected number of incidental correlation predicted by the simulations.

## Supplementary Materials

The following are available online at https://www.mdpi.com/article/10.3390/ijms22158240/s1.
