# Supplementary material for: Direct Measurement of the Affinity between tBid and Bax in a Mitochondria-Like Membrane
Source: Int J Mol Sci. 2021 Jul 31;22(15):8240. doi: 10.3390/ijms22158240 (PMC8348223; doi:10.3390/ijms22158240)
Supplement: Supplementary file 1 [file ijms-22-08240-s001.zip › ijms-1272431-supplementary.pdf]

Supplemental Material: Direct Measurement of the Affinity between Bid and Bax in a Mitochondria-Like Membrane

Markus Rose, Martin Kurylowicz, Mohammad Mahmood, Sheldon Winkel, Jose Moran-Mirabal, Cécile Fradin

1 Relationship between 3D and 2D equilibrium constants

When single conformational ensembles are considered for Bax and tBid species (total concentration $[Bax]$ and $[tBid]$), and for tBid-Bax complexes (concentration $[tBid - Bax]$), regardless of whether they are soluble or associated with membranes, the equilibrium between these ensembles is characterized by an apparent dissociation constant, K_D^{app}:

$$K_D^{app} = \frac{[Bid].[Bax]}{[tBid - Bax]}. \tag{1}$$

In reality, Bax species are divided into soluble and membrane species, and the equilibrium between these two subpopulations is regulated by a partition coefficient, P_{Bax}:

$$P_{Bax} = \frac{n_{Bax}^m / V_m}{n_{Bax}^s / V_s}, \tag{2}$$

where n_{Bax}^m and n_{Bax}^s are the number of Bax molecules in the membrane and soluble fractions, and V_m and V_s are the volumes of the membrane and solution. Since the number of Bax molecules is conserved, we have $n_{Bax} = n_{Bax}^m + n_{Bax}^s$, and the total Bax concentration can be expressed as a function of n_{Bax}^m:

$$[Bax] = \frac{n_{Bax}}{V} = \frac{n_{Bax}^m + n_{Bax}^s}{V} = \left(\frac{1}{P_{Bax}} \frac{V}{V_m} + 1\right) \frac{n_{Bax}^m}{V} \tag{3}$$

where we assume that the volume of the soluble phase can be assimilated to the total volume of the sample: $V_s \approx V$. A similar equation can be written for tBid species, which are also subdivided between membrane species and soluble species.

In contrast to what happens for tBid and Bax species, tBid-Bax complexes are not detected in the absence of a membrane, so we assume that all the tBid-Bax complexes are membrane-bound. Consequently:

$$[tBid - Bax] = \frac{n_{tBid-Bax}^m}{V}. \tag{4}$$

The apparent K_D^{app} can then be expressed as a function of the number of membrane-bound molecules:

$$K_D^{app} = \frac{n_{tBid}^m n_{Bax}^m}{n_{tBid-Bax}^m} \frac{1}{V} \left(\frac{1}{P_{tBid}} \frac{V}{V_m} + 1\right) \left(\frac{1}{P_{Bax}} \frac{V}{V_m} + 1\right). \tag{5}$$

The surface concentration of the proteins (denoted c) is obtained by dividing the number of membrane bound proteins by the total surface area of the membrane, $S_M = V_m/d$ (where d is the membrane thickness. For example, for Bax:

$$c_{Bax} = \frac{n_{Bax}^m}{S_M} = \frac{n_{Bax}^m d}{V_M} \tag{6}$$

Thus Eq. 5 can be rewritten as:

$$K_D^{app} = \frac{c^m_{tBid}.c^m_{Bax}}{c^m_{tBid-Bax}} \frac{1}{d} \frac{V_m}{V} \left(\frac{1}{P_{tBid}} \frac{V}{V_m} + 1 \right) \left(\frac{1}{P_{Bax}} \frac{V}{V_m} + 1 \right). \tag{7}$$

The $2D - K_D$ associated with the formation of the tBid-Bax complex at the membrane (from membrane-bound tBid and Bax) is defined as:

$$2D - K_D = \frac{c^m_{tBid}.c^m_{Bax}}{c^m_{tBid-Bax}}. \tag{8}$$

Thus Eq. 9 can be rewritten as:

$$K_D^{app} = 2D - K_D \times \frac{1}{d} \frac{V_m}{V} \left(\frac{1}{P_{tBid}} \frac{V}{V_m} + 1 \right) \left(\frac{1}{P_{Bax}} \frac{V}{V_m} + 1 \right). \tag{9}$$

Finally, in order to clarify the dependence of K_D^{app} on the lipid concentration, we note that $V_m = [L]v_L V$, where $[L]$ is the lipid concentration, and v_L the volume of a lipid. This leads to:

$$K_D^{app} = 2D - K_D \times \frac{[L]v_L}{d} \left(\frac{1}{[L]v_L P_{tBid}} + 1 \right) \left(\frac{1}{[L]v_L P_{Bax}} + 1 \right). \tag{10}$$

In cases where the lipid concentration is sufficiently small (as certainly happens in the case of supported lipid bilayers), we have $[L]v_L P \ll 1$ for both tBid and Bax, and Eq. 10 then simplifies into:

$$K_D^{app} = 2D - K_D \times \frac{1}{d[L]v_L P_{tBid} P_{Bax}}. \tag{11}$$

The dependence of the measured K_D^{app} on lipid concentration is plotted below in Fig. S1, assuming that $2D - K_D = 1\,\mu\text{m}^{-2}$, that the membrane thickness is $d = 4\,\text{nm}$ and that the molar volume of the lipids is $v_L = 7.6 \times 10^{-4}$ l/mol.

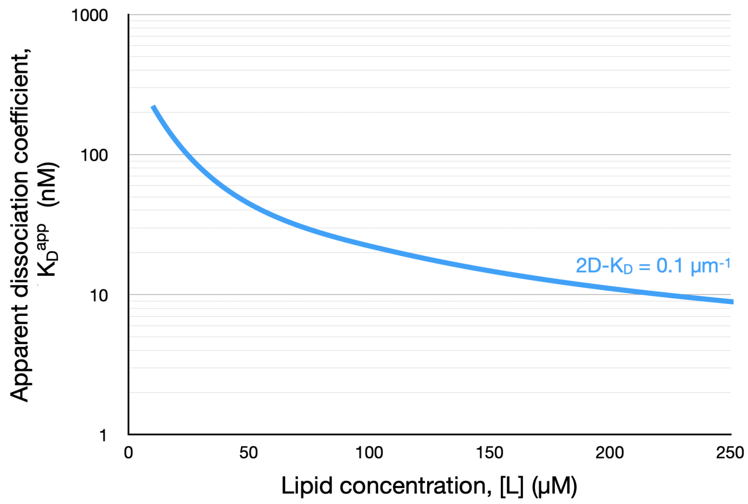

Figure S1: Apparent dissociation coefficient as a function of lipid concentration. Apparent dissociation coefficient, K_D^{app}, which would be measured considering all protein species as soluble species, for an actual two-dimensional dissociation coefficient $2D - K_D = 1\,\mu\text{m}^{-2}$ and partition coefficients $P_{tBid} = 7000$ and $P_{Bax} = 3500$, as a function of lipid concentration. The calculation was done using Eq. 11 and assuming that the membrane thickness is $d = 4\,\text{nm}$ and that the molar volume of the lipids is $v_L = 7.6 \times 10^{-4}$ l/mol.

2 Supplemental Figures

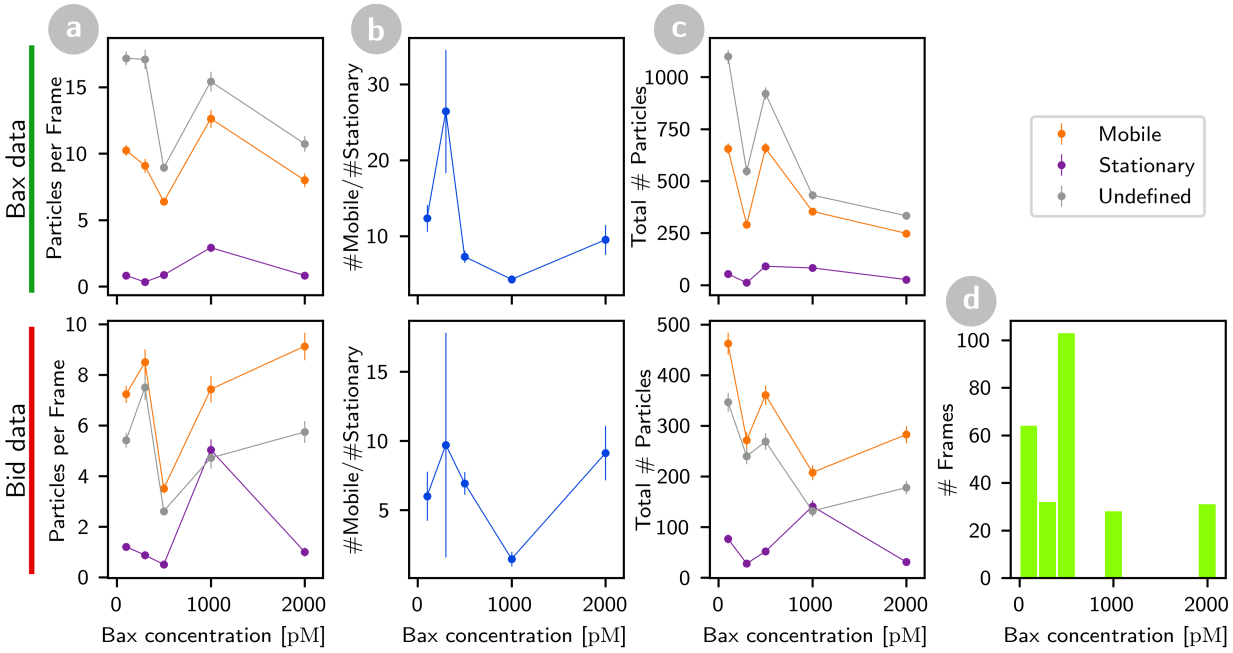

Figure S2: Detected particles as a function of Bax concentration. (a) The number of detected particles per frame is given for the range of Bax concentrations 0.1 nM - 2 nM. The detection can be either stationary, mobile or undefined. (b) Similarly the ratio of mobile to stationary particles throughout the Bax concentration range is provided in (b). (c) shows the total number of detected particles for a given Bax concentrations and (d) the number of frames at each concentration.

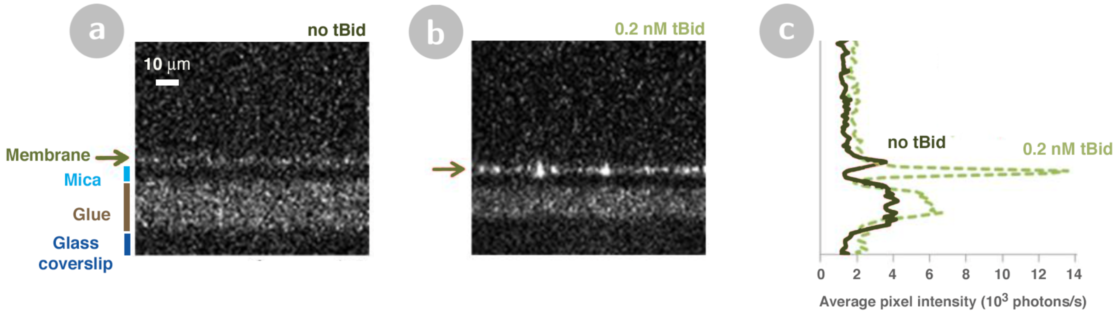

Figure S3: Binding of Bax-HiLyte488 to supported lipid bilayers. (a,b) Confocal images of a supported lipid bilayer incubated with 2 nM Bax-Hilyte488 for 1 hour at 37 °C (488 nm excitation) either in the absence (a) or in the presence (b) of 0.2 nM tBid. The signal observed is due to both the fluorescence of Bax-Hilyte488 and the autofluorescence of the glue used to fix the mica on the glass coverslip. The approximate position of the mica coverslip and of the glue layer are indicated. Imaging conditions: pixel size 1 µm, pixel dwell time 1 ms, laser power 5 µW. (C) Average intensity profile along the optical axis of the images in (a,b). The average pixel intensity at the plane of the membrane was 14 kHz in the presence of tBid and 4 kHz in its absence.

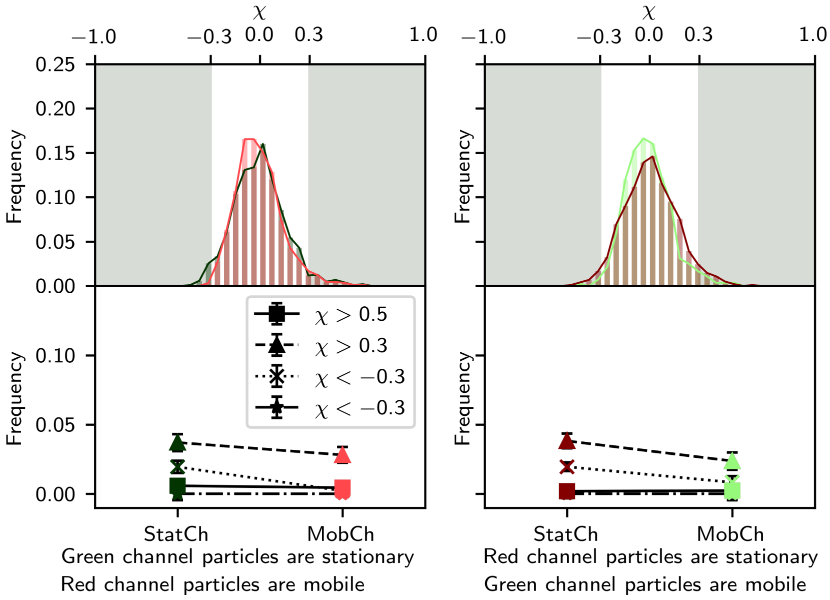

Figure S4: Cross-correlation coefficient of simulated unbound particles with different mobilities in each channel. The left panel represents stationary particles in the green channel and mobile particles in the red channel, while the right panel has the mobile particles in the green channel and the stationary particles in the red channel. Each channel was simulated with particle concentrations of 0.2 particles/µm^2. The green channel has $w_0 = 320$ nm and the red channel has $w_0 = 370$ nm.

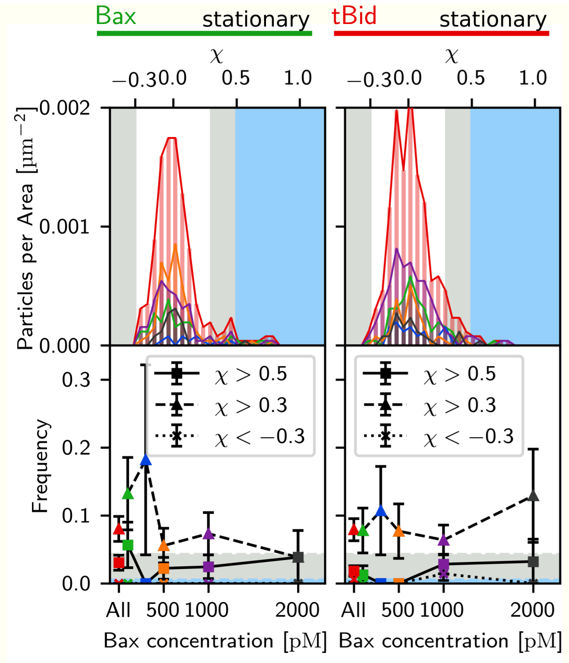

Figure S5: Particle cross-correlation coefficients measured for stationary and mobile tBid and Bax oligomers as a function of Bax concentration. The upper panels show the cross-correlation coefficient distributions for all experimental Bax starting concentrations. The grey areas correspond to slight cross- or anti-correlation ($0.3 < \chi < 0.5$ or $-0.5 < \chi < -0.3$) and the blue area highlights strong cross-correlation ($\chi > 0.5$). The lower panels display the relative particle counts in each oligomer class when correlated ($\chi > 0.3$ and $\chi > 0.5$) and anti-correlated ($\chi < -0.3$). The highlighted areas show the absolute maximum amount of accidental positive correlations (blue for $\chi > 0.5$, grey for $\chi > 0.3$) to be expected given a particle concentration similar to that observed in experiments (0.05 particles/μm^2 for stationary particles and 0.15 particles/μm^2 for mobile particles).
